# Author Correction: Identification of multiple risk loci and regulatory mechanisms influencing susceptibility to multiple myeloma

**DOI:** 10.1038/s41467-018-08107-8

**Published:** 2019-01-10

**Authors:** Molly Went, Amit Sud, Asta Försti, Britt-Marie Halvarsson, Niels Weinhold, Scott Kimber, Mark van Duin, Gudmar Thorleifsson, Amy Holroyd, David C. Johnson, Ni Li, Giulia Orlando, Philip J. Law, Mina Ali, Bowang Chen, Jonathan S. Mitchell, Daniel F. Gudbjartsson, Rowan Kuiper, Owen W. Stephens, Uta Bertsch, Peter Broderick, Chiara Campo, Obul R Bandapalli, Hermann Einsele, Walter A. Gregory, Urban Gullberg, Jens Hillengass, Per Hoffmann, Graham H. Jackson, Karl-Heinz Jöckel, Ellinor Johnsson, Sigurður Y. Kristinsson, Ulf-Henrik Mellqvist, Hareth Nahi, Douglas Easton, Paul Pharoah, Alison Dunning, Julian Peto, Federico Canzian, Anthony Swerdlow, Rosalind A. Eeles, Zsofia Kote-Jarai, Kenneth Muir, Nora Pashayan, The PRACTICAL consortium, Brian E. Henderson, Christopher A. Haiman, Sara Benlloch, Fredrick R. Schumacher, Ali Amin Al Olama, Sonja I. Berndt, David V. Conti, Fredrik Wiklund, Stephen Chanock, Victoria L. Stevens, Catherine M. Tangen, Jyotsna Batra, Judith Clements, Henrik Gronberg, Johanna Schleutker, Demetrius Albanes, Stephanie Weinstein, Alicja Wolk, Catharine West, Lorelei Mucci, Géraldine Cancel-Tassin, Stella Koutros, Karina Dalsgaard Sorensen, Eli Marie Grindedal, David E. Neal, Freddie C. Hamdy, Jenny L. Donovan, Ruth C. Travis, Robert J. Hamilton, Sue Ann Ingles, Barry Rosenstein, Yong-Jie Lu, Graham G. Giles, Adam S. Kibel, Ana Vega, Manolis Kogevinas, Kathryn L. Penney, Jong Y. Park, Janet L. Stanford, Cezary Cybulski, Børge G. Nordestgaard, Hermann Brenner, Christiane Maier, Jeri Kim, Esther M. John, Manuel R. Teixeira, Susan L. Neuhausen, Kim De Ruyck, Azad Razack, Lisa F. Newcomb, Davor Lessel, Radka Kaneva, Nawaid Usmani, Frank Claessens, Paul A. Townsend, Manuela Gago-Dominguez, Monique J. Roobol, Florence Menegaux, Kay-Tee Khaw, Lisa Cannon-Albright, Hardev Pandha, Stephen N. Thibodeau, Jolanta Nickel, Markus M. Nöthen, Thorunn Rafnar, Fiona M. Ross, Miguel Inacio da Silva Filho, Hauke Thomsen, Ingemar Turesson, Annette Vangsted, Niels Frost Andersen, Anders Waage, Brian A. Walker, Anna-Karin Wihlborg, Annemiek Broyl, Faith E. Davies, Unnur Thorsteinsdottir, Christian Langer, Markus Hansson, Hartmut Goldschmidt, Martin Kaiser, Pieter Sonneveld, Kari Stefansson, Gareth J. Morgan, Kari Hemminki, Björn Nilsson, Richard S. Houlston

**Affiliations:** 1 Division of Genetics and Epidemiology, The Institute of Cancer Research, London, SW7 3RP UK; 2 German Cancer Research Center, 69120 Heidelberg, Germany; 3 Center for Primary Health Care Research, Lund University, SE-205 02 Malmo, Sweden; 4 Hematology and Transfusion Medicine, Department of Laboratory Medicine, BMC B13, Lund University, SE-221 84 Lund, Sweden; 5 Myeloma Institute for Research and Therapy, University of Arkansas for Medical Sciences, Little Rock, AR 72205 USA; 6 Department of Internal Medicine V, University of Heidelberg, 69117 Heidelberg, Germany; 7 Division of Molecular Pathology, The Institute of Cancer Research, London, SW7 3RP UK; 8 Department of Hematology, Erasmus MC Cancer Institute, 3075 EA Rotterdam, The Netherlands; 9 deCODE Genetics, Sturlugata 8, IS-101 Reykjavik, Iceland; 10 School of Engineering and Natural Sciences, University of Iceland, IS-101 Reykjavik, Iceland; 11 National Centre of Tumor Diseases, 69120 Heidelberg, Germany; 12 University Clinic of Würzburg, 97080 Würzburg, Germany; 13 Clinical Trials Research Unit, University of Leeds, Leeds, LS2 9PH UK; 14 Institute of Human Genetics, University of Bonn, D-53127 Bonn, Germany; 15 Division of Medical Genetics, Department of Biomedicine, University of Basel, 4003 Basel, Switzerland; 16 Royal Victoria Infirmary, Newcastle upon Tyne, NE1 4LP UK; 17 Institute for Medical Informatics, Biometry and Epidemiology, University Hospital Essen, University of Duisburg–Essen, Essen, D-45147 Germany; 18 Department of Hematology, Landspitali, National University Hospital of Iceland, IS-101 Reykjavik, Iceland; 19 Section of Hematology, Sahlgrenska University Hospital, Gothenburg, 413 45 Sweden; 20 Center for Hematology and Regenerative Medicine, SE-171 77 Stockholm, Sweden; 21 Centre for Cancer Genetic Epidemiology, Department of Oncology, University of Cambridge, Cambridge, CB1 8RN UK; 22 Centre for Cancer Genetic Epidemiology, Department of Public Health and Primary Care, University of Cambridge, Cambridge, CB1 8RN UK; 23 Department of Non-Communicable Disease Epidemiology, London School of Hygiene and Tropical Medicine, London, WC1E 7HT UK; 24 Genomic Epidemiology Group, German Cancer Research Center (DKFZ), Heidelberg, 69120 Germany; 25 Division of Breast Cancer Research, The Institute of Cancer Research, London, SW7 3RP UK; 26 Royal Marsden NHS Foundation Trust, Fulham Road, London, SW3 6JJ UK; 27 Institute of Population Health, University of Manchester, Manchester, M13 9PL UK; 28 Warwick Medical School, University of Warwick, Coventry, CV4 7AL UK; 29 Department of Applied Health Research, University College London, London, WC1E 7HB UK; 30 Department of Genomics, Life & Brain Center, University of Bonn, D-53127 Bonn, Germany; 31 Wessex Regional Genetics Laboratory, University of Southampton, Salisbury, SP2 8BJ UK; 32 Hematology Clinic, Skåne University Hospital, SE-221 85 Lund, Sweden; 33 Department of Haematology, University Hospital of Copenhagen at Rigshospitalet, Blegdamsvej 9, DK-2100 Copenhagen, Denmark; 34 Department of Haematology, Aarhus University Hospital, Tage-Hansens Gade 2, DK-8000 Aarhus C, Denmark; 35 Department of Cancer Research and Molecular Medicine, Norwegian University of Science and Technology, Box 8905, N-7491 Trondheim, Norway; 36 Faculty of Medicine, University of Iceland, IS-101 Reykjavik, Iceland; 37 Department of Internal Medicine III, University of Ulm, D-89081 Ulm, Germany; 38 Broad Institute, 7 Cambridge Center, Cambridge, MA 02142 USA; 39 Department of Preventive Medicine, Keck School of Medicine, University of Southern California/Norris Comprehensive Cancer Center, Los Angeles, 90033 CA USA; 40 Department of Epidemiology and Biostatistics, Case Western Reserve University, Cleveland, 44106 OH USA; 41 Seidman Cancer Center, University Hospitals, Cleveland, 44106 OH USA; 42 Department of Clinical Neurosciences, University of Cambridge, Cambridge, CB2 1TN UK; 43 Division of Cancer Epidemiology and Genetics, National Cancer Institute, NIH, Bethesda, 20892 MD USA; 44 Department of Medical Epidemiology and Biostatistics, Karolinska Institute, Stockholm, SE-177 77 Sweden; 45 Epidemiology Research Program, American Cancer Society, 250 Williams Street, Atlanta, 30303 GA USA; 46 Australian Prostate Cancer Research Centre-Qld, Institute of Health and Biomedical Innovation and School of Biomedical Science, Queensland University of Technology, Brisbane, 4059 QLD Australia; 47 Translational Research Institute, Brisbane, 4102 QLD Australia; 48 Department of Medical Biochemistry and Genetics, Institute of Biomedicine, University of Turku, Turku, FI-20520 Finland; 49 Tyks Microbiology and Genetics, Department of Medical Genetics, Turku University Hospital, Turku, FI-20520 Finland; 50 BioMediTech, University of Tampere, Tampere, 33100 Finland; 51 Division of Nutritional Epidemiology, Institute of Environmental Medicine, Karolinska Institutet, Solna, SE-171 77 Sweden; 52 Institute of Cancer Sciences, University of Manchester, Manchester Academic Health Science Centre, Radiotherapy Related Research, The Christie Hospital NHS Foundation Trust, Manchester, M13 9NT UK; 53 Department of Epidemiology, Harvard School of Public Health, Boston, 02115 MA USA; 54 CeRePP, Pitie-Salpetriere Hospital, Paris, 75013 France; 55 UPMC Univ Paris 06, GRC No. 5 ONCOTYPE-URO, CeRePP, Tenon Hospital, Paris, 75020 France; 56 Department of Molecular Medicine, Aarhus University Hospital, Aarhus, 8000 Denmark; 57 Department of Clinical Medicine, Aarhus University, Aarhus, 8000 Denmark; 58 Department of Medical Genetics, Oslo University Hospital, Oslo, N-0424 Norway; 59 University of Cambridge, Department of Oncology, Addenbrooke’s Hospital, Cambridge, CB2 0QQ UK; 60 Cancer Research UK Cambridge Research Institute, Ka Shing Centre, Cambridge, CB2 0RE UK; 61 Nuffield Department of Surgical Sciences, University of Oxford, Oxford, OX3 9DU UK; 62 Faculty of Medical Science, University of Oxford, John Radcliffe Hospital, Oxford, OX3 9DU UK; 63 School of Social and Community Medicine, University of Bristol, Bristol, BS8 2PS UK; 64 Cancer Epidemiology, Nuffield Department of Population Health, University of Oxford, Oxford, OX3 7LF UK; 65 Department of Surgical Oncology, Princess Margaret Cancer Centre, Toronto, M5G 2M9 Canada; 66 Department of Radiation Oncology, Icahn School of Medicine at Mount Sinai, New York, 10029 NY USA; 67 Department of Genetics and Genomic Sciences, Icahn School of Medicine at Mount Sinai, New York, NY 10029 USA; 68 Centre for Molecular Oncology, Barts Cancer Institute, Queen Mary University of London, John Vane Science Centre, London, EC1M 6BQ UK; 69 Cancer Epidemiology & Intelligence Division, The Cancer Council Victoria, Melbourne, 3004 VIC Australia; 70 Centre for Epidemiology and Biostatistics, Melbourne School of Population and Global Health, The University of Melbourne, Melbourne, 3053 VIC Australia; 71 Division of Urologic Surgery, Brigham and Womens Hospital, Boston, 02115 MA USA; 72 Fundación Pública Galega de Medicina Xenómica-SERGAS, Grupo de Medicina Xenómica, CIBERER, IDIS, Santiago de Compostela, 15782 Spain; 73 Centre for Research in Environmental Epidemiology (CREAL), Barcelona Institute for Global Health (ISGlobal), Barcelona, 60803 Spain; 74 CIBER Epidemiología y Salud Pública (CIBERESP), Madrid, 28029 Spain; 75 IMIM (Hospital del Mar Research Institute), Barcelona, 08003 Spain; 76 Universitat Pompeu Fabra (UPF), Barcelona, 08002 Spain; 77 Channing Division of Network Medicine, Department of Medicine, Brigham and Women’s Hospital/Harvard Medical School, Boston, 02115 MA USA; 78 Department of Cancer Epidemiology, Moffitt Cancer Center, Tampa, 33612 FL USA; 79 Division of Public Health Sciences, Fred Hutchinson Cancer Research Center, Seattle, 98109 WA USA; 80 Department of Epidemiology, School of Public Health, University of Washington, Seattle, 98195 WA USA; 81 International Hereditary Cancer Center, Department of Genetics and Pathology, Pomeranian Medical University, Szczecin, 70-001 Poland; 82 Faculty of Health and Medical Sciences, University of Copenhagen, Copenhagen, 1165 Denmark; 83 Department of Clinical Biochemistry, Herlev and Gentofte Hospital, Copenhagen University Hospital, Herlev, 2900 Denmark; 84 Division of Clinical Epidemiology and Aging Research, German Cancer Research Center (DKFZ), Heidelberg, 69120 Germany; 85 German Cancer Consortium (DKTK), German Cancer Research Center (DKFZ), Heidelberg, 69120 Germany; 86 Division of Preventive Oncology, German Cancer Research Center (DKFZ) and National Center for Tumor Diseases (NCT), Heidelberg, 69120 Germany; 87 Institute for Human Genetics, University Hospital Ulm, Ulm, 89081 Germany; 88 Department of Genitourinary Medical Oncology, The University of Texas M. D. Anderson Cancer Center, Houston, 77030 TX USA; 89 Cancer Prevention Institute of California, Fremont, 94538 CA USA; 90 Department of Health Research & Policy (Epidemiology) and Stanford Cancer Institute, Stanford University School of Medicine, Stanford 94305, CA USA; 91 Department of Genetics, Portuguese Oncology Institute of Porto, Porto, 4200-072 Portugal; 92 Biomedical Sciences Institute (ICBAS), University of Porto, Porto, 4200-072 Portugal; 93 Department of Population Sciences, Beckman Research Institute of the City of Hope, Duarte, 91016 CA USA; 94 Ghent University, Faculty of Medicine and Health Sciences, Basic Medical Sciences, Ghent, 9000 Belgium; 95 Department of Surgery, Faculty of Medicine, University of Malaya, Kuala Lumpur, 50603 Malaysia; 96 Department of Urology, University of Washington, Seattle 98105, WA USA; 97 Institute of Human Genetics, University Medical Center Hamburg-Eppendorf, Hamburg, 20246 Germany; 98 Molecular Medicine Center, Department of Medical Chemistry and Biochemistry, Medical University, Sofia, 1431 Bulgaria; 99 Department of Oncology, Cross Cancer Institute, University of Alberta, Edmonton, T6G 2R3 Alberta Canada; 100 Division of Radiation Oncology, Cross Cancer Institute, Edmonton, T6G 1Z2 AB Canada; 101 Molecular Endocrinology Laboratory, Department of Cellular and Molecular Medicine, KU Leuven, Leuven, 3000 Belgium; 102 Institute of Cancer Sciences, Manchester Cancer Research Centre, University of Manchester, Manchester Academic Health Science Centre, St. Mary’s Hospital, Manchester, M13 9WL UK; 103 Genomic Medicine Group, Galician Foundation of Genomic Medicine, Instituto de Investigacion Sanitaria de Santiago de Compostela (IDIS), Complejo Hospitalario Universitario de Santiago, Servicio Galego de Saúde, SERGAS, Santiago De Compostela, 15706 Spain; 104 University of California San Diego, Moores Cancer Center, La Jolla, 92093 CA USA; 105 Department of Urology, Erasmus University Medical Center, Rotterdam, 3015 The Netherlands; 106 Cancer & Environment Group, Center for Research in Epidemiology and Population Health (CESP), INSERM, University Paris-Sud, University Paris-Saclay, Villejuif, F-94805 France; 107 Clinical Gerontology Unit, University of Cambridge, Cambridge, CB2 2QQ UK; 108 Division of Genetic Epidemiology, Department of Medicine, University of Utah School of Medicine, Salt Lake City, 84132 UT USA; 109 George E. Wahlen Department of Veterans Affairs Medical Center, Salt Lake City, 84148 UT USA; 110 The University of Surrey, Guildford, Surrey GU2 7XH UK

Correction to: *Nature Communications*; 10.1038/s41467-018-04989-w, published online 13 September 2018

The original version of this Article contained an error in the spelling of a member of the PRACTICAL Consortium, Manuela Gago-Dominguez, which was incorrectly given as Manuela Gago Dominguez. This has now been corrected in both the PDF and HTML versions of the Article. Furthermore, in the original HTML version of this Article, the order of authors within the author list was incorrect. The PRACTICAL consortium was incorrectly listed after Richard S. Houlston and should have been listed after Nora Pashayan. This error has been corrected in the HTML version of the Article; the PDF version was correct at the time of publication.

