# Supplementary material for: Identification of multiple risk loci and regulatory mechanisms influencing susceptibility to multiple myeloma
Source: Nat Commun. 2018 Sep 13;9:3707. doi: 10.1038/s41467-018-04989-w (PMC6137048; doi:10.1038/s41467-018-04989-w)
Supplement: Supplementary file 1 — Supplementary Information [file 41467_2018_4989_MOESM1_ESM.docx]

**SUPPLEMENTARY INFORMATION**

**Identification of multiple risk loci and regulatory mechanisms influencing susceptibility to multiple myeloma**

Went *et al.*

**Supplementary Table 1: Details of the quality control filters applied to each GWAS.** Samples were excluded due to call rate (<95% or failed genotyping), ancestry (principle components analysis or other samples reported to be not of white, European descent), relatedness (any individuals found to be duplicated or related within or between data sets through IBS) or sex discrepancy. Dutch, German, UK, USA, Sweden/Norway and Iceland: These studies have been previously reported in their entirety with comprehensive details on QC^1-4^. MyIX, Myeloma IX; MyXI, Myeloma XI; B-PROOF, B-vitamins for the prevention of osteoporotic fractures; UKGPCS, UK Genetic Prostate Cancer Study; BCAC, Breast Cancer Association Consortium.

**Supplementary Table 2: Details of the quality control filters applied to each GWAS.** For the OncoArray genotyped SNPs with a call rate <95% were excluded as were those with a MAF <0.01 or showing significant deviation from Hardy-Weinberg equilibrium (*i.e*. *P* < 10^-5^). Imputed SNPs with information score <0.8 and MAF <0.01 were excluded. Dutch, German, UK, USA, Sweden/Norway and Iceland: These studies have been previously reported in their entirety with comprehensive details on QC^1-4^.

**Supplementary Table 3: Details of the replication sample recruitment.**

(Table continued on following page)

(Table continued on following page)

**Supplementary Table 4: Summary statistics for novel variants showing an association with multiple myeloma risk in the GWAS meta-analysis at *P* < 1.0 × 10^-6^.** These were taken forward for replication and those which showed association at *P* < 5× 10^-8^ were considered genome-wide significant. Odds ratios derived with respect to the risk allele. Cases RAF, risk allele frequency in discovery cases. Controls RAF, risk allele frequency in discovery controls. Shown are discovery association *P* values for individual studies. Meta_GWAS_ shows a meta-analysis of previously published GWAS including new discovery OncoArray dataset. Meta_GWAS+REP_ represents a meta-analysis of previously published GWAS, new discovery OncoArray dataset and replication series. Heterogeneity index, *I^2^* (0-100), quantifies the proportion of the total variation due to heterogeneity.

**Supplementary Table 5: Replication of top association signals.** Showing SNPs which were taken forward for replication genotyping. Cases RAF, risk allele frequency of replication cases; Control RAF, risk allele frequency of replication controls. *P* values are shown for each replication series (logistic regression). rs17507636 had been previously replicated in the German cohort, with association values^4^; cases RAF: 0.760, controls RAF: 0.735, OR: 1.15, *P* value: 0.06. A meta-analysis of this with discovery cohorts and replication series was performed using R version 3.3.1 (R Development Core Team, Vienna, Austria).

**Supplementary Table 6: Relationship between SNP genotype and sex**. Analysis based on beta values calculated from logistic regression on the discovery phase data sets from UK (2282 cases), Oncoarray (878 cases), German (1508 cases) and USA (780 cases) series. The meta-analysis was conducted using a fixed-effects model. This assumes that the underlying effect across all studies is the same. To test for potential heterogeneity, Cochran’s Q-statistic was calculated such that *P_HET_* >0.05 implied the presence of non-significant heterogeneity. The heterogeneity index, *I^2^* (0-100), was also measured; this quantifies the proportion of the total variation due to heterogeneity.

**Supplementary Table 7:** **Relationship between SNP genotype and age at diagnosis.** Analysis based on beta values calculated from linear regression on the discovery phase data sets from UK (2282 cases), Oncoarray (878 cases), German (1508 cases) and USA (780 cases) cohorts. The meta-analysis was conducted using a fixed-effects model. This assumes that the underlying effect across all studies is the same. To test for potential heterogeneity, Cochran’s Q-statistic was calculated such that *P_HET_* >0.05 implied the presence of non-significant heterogeneity. The heterogeneity index, *I^2^* (0-100), was also measured; this quantifies the proportion of the total variation due to heterogeneity.

**Supplementary Tables 8: Relationship between SNP genotype and t(4;14) subtype.** German cases: 142, UK cases: 170, Oncoarray cases: 33, Meta: 345. Case-only analysis; Beta values obtained from logistic regression. FISH and ploidy classification of UK and German samples were determined as previously described^5,6^.

**Supplementary Tables 9:** **Relationship between SNP genotype and t(11;14) subtype**. German cases: 277, UK cases: 231, Oncoarray cases: 47, Meta: 555. Case-only analysis; Beta values obtained from logistic regression. FISH and ploidy classification of UK and German samples were determined as previously described^5,6^.

**Supplementary Tables 10:** **Relationship between SNP genotype and t(14;16) subtype.** German cases: 29, UK cases: 24, Oncoarray cases: 8, Meta: 61 Case-only analysis; Beta values obtained from logistic regression. FISH and ploidy classification of UK and German samples were determined as previously described^5,6^.

**Supplementary Tables 11:** **Relationship between SNP genotype and hyperdiploid subtype**. German cases: 661, UK cases: 702, Oncoarray cases: 257, Meta: 1,620. Case-only analysis; Beta values obtained from logistic regression. FISH and ploidy classification of UK and German samples were determined as previously described^5,6^.

**Supplementary Table 12: Relationship between genome-wide significant SNPs genotype and patient overall survival^7^.** Data from: 1,165 cases from the UK MRC Myeloma-IX trial (UK-MyIX); 877 MM cases from the UK MRC Myeloma-XI trial (UK-MyXI); 511 of the patients recruited to the German-GWAS (GER-GMMG); 703 MM cases in the UAMS Myeloma Institute for Research and Therapy GWAS (US-UAMS). *P*-values calculated from Cox regression analysis. Data for SNPs rs2811710, rs7577599 and rs138747, or a correlated SNP (*r^2^* >0.6) to use as proxy, were not present in the survival analysis.

**Supplementary Table 13: Concordance between directly sequenced and imputed genotype. Showing SNPs which were genome-wide significant after replication.** These comprised 147 randomly selected samples from the Oncoarray case series. AA, major homozygote; Aa, heterozygote; aa, minor homozygote. *r^2^* indicates Pearson product-moment correlation coefficient between imputed and sequenced genotype.

**Supplementary Table 14: Details of genotyping primers and reaction conditions.**

**Supplementary Table 15: Details of sequencing primers and reaction conditions.**

**KASPAR conditions**

**Std42**

- Hot Start: 94ºC for 15 minutes

- Stage 1: 20 cycles

o 94ºC for 10 seconds

o 57ºC for 5 seconds

o 72ºC for 10 seconds

- Stage 2: 22 cycles

o 94ºC for 10 seconds

o 57ºC for 20 seconds

o 72ºC for 40 seconds

**Std42plus5**

- Hot Start: 94ºC for 15 minutes

- Stage 1: 20 cycles

o 94ºC for 10 seconds

o 57ºC for 5 seconds

o 72ºC for 10 seconds

- Stage 2: 22 cycles

o 94ºC for 10 seconds

o 57ºC for 20 seconds

o 72ºC for 40 seconds

- Stage 3: 5 cycles

o 94ºC for 10 seconds

o 57ºC for 1 minute

**Std42plus10**

- Hot Start: 94ºC for 15 minutes

- Stage 1: 20 cycles

o 94ºC for 10 seconds

o 57ºC for 5 seconds

o 72ºC for 10 seconds

- Stage 2: 22 cycles

o 94ºC for 10 seconds

o 57ºC for 20 seconds

o 72ºC for 40 seconds

- Stage 3: 10 cycles

o 94ºC for 10 seconds

o 57ºC for 1 minute

**Std42plus15**

- Hot Start: 94ºC for 15 minutes

- Stage 1: 20 cycles

o 94ºC for 10 seconds

o 57ºC for 5 seconds

o 72ºC for 10 seconds

- Stage 2: 22 cycles

o 94ºC for 10 seconds

o 57ºC for 20 seconds

o 72ºC for 40 seconds

- Stage 3: 15 cycles

o 94ºC for 10 seconds

o 57ºC for 1 minute

**Sequencing conditions**

**Std**

- 95ºC for 5 minutes

- 25 cycles

o 96ºC for 30 seconds

o 50ºC for 15 seconds

o 60ºC for 1 minute

**Supplementary Table 16: Summary of results from SMR analysis.** We set a threshold for the SMR test of *P_SMR_* <1×10^-3^ corresponding to a Bonferroni correction for 45 tests. For all genes passing this threshold we generated plots of the eQTL and GWAS associations at the locus, as well as plots of GWAS and eQTL effect sizes (i.e. corresponding to input for the HEIDI heterogeneity test). HEIDI test *P* values <0.05 were considered as being reflective of heterogeneity. This threshold is conservative for gene discovery because it retains fewer genes than when correcting for multiple testing. Probes which passed the HEIDI threshold are highlighted in grey.

**Supplementary Table 17: Full lists of TF binding at selected loci.** TF ChIP-seq (161 factors) with Factorbook Motifs for GM12878 were downloaded from ENCODE^8^.


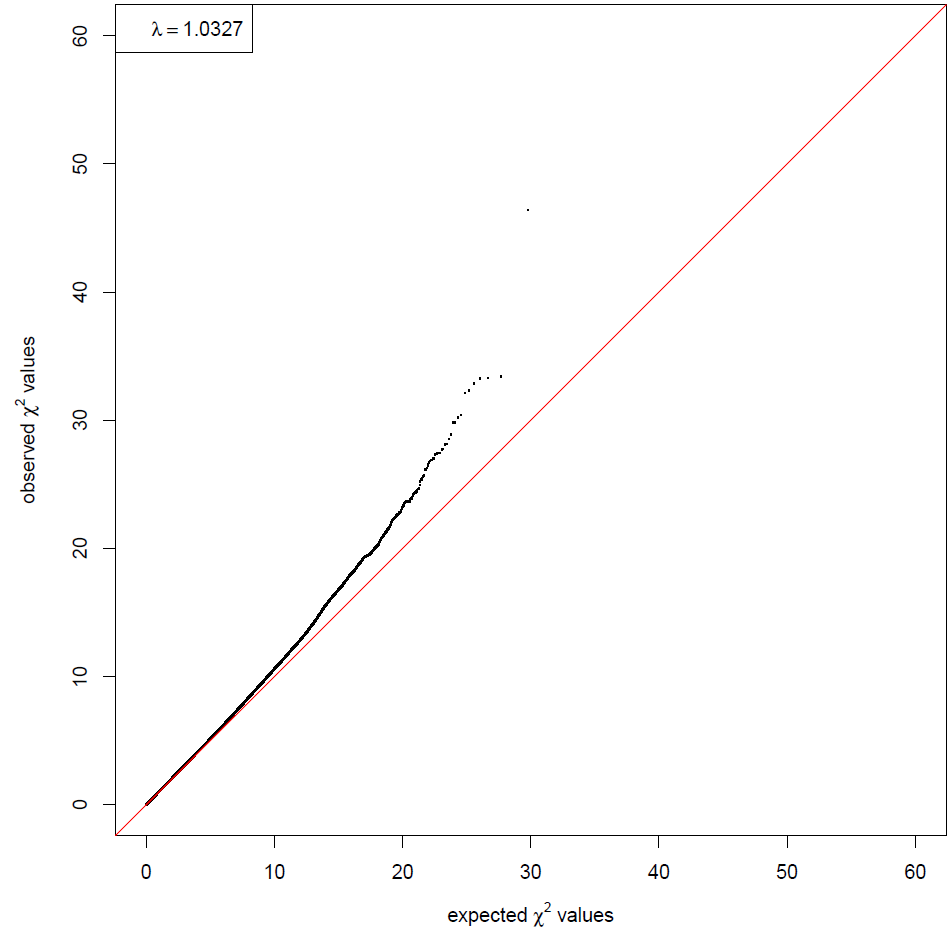


Supplementary Figure 1: Quantile-Quantile (*Q-Q*) plots of observed and expected χ^2^ values of association between SNP genotype and risk of multiple myeloma after imputation for the OncoArray cohort. λ=1.0327, λ_1000_=1.0209. The red line represents the null hypothesis of no true association. *Q-Q* plots for the UK, Sweden/Norway, Germany, Iceland, USA and Netherlands sets have been previously reported^1-4^.

**
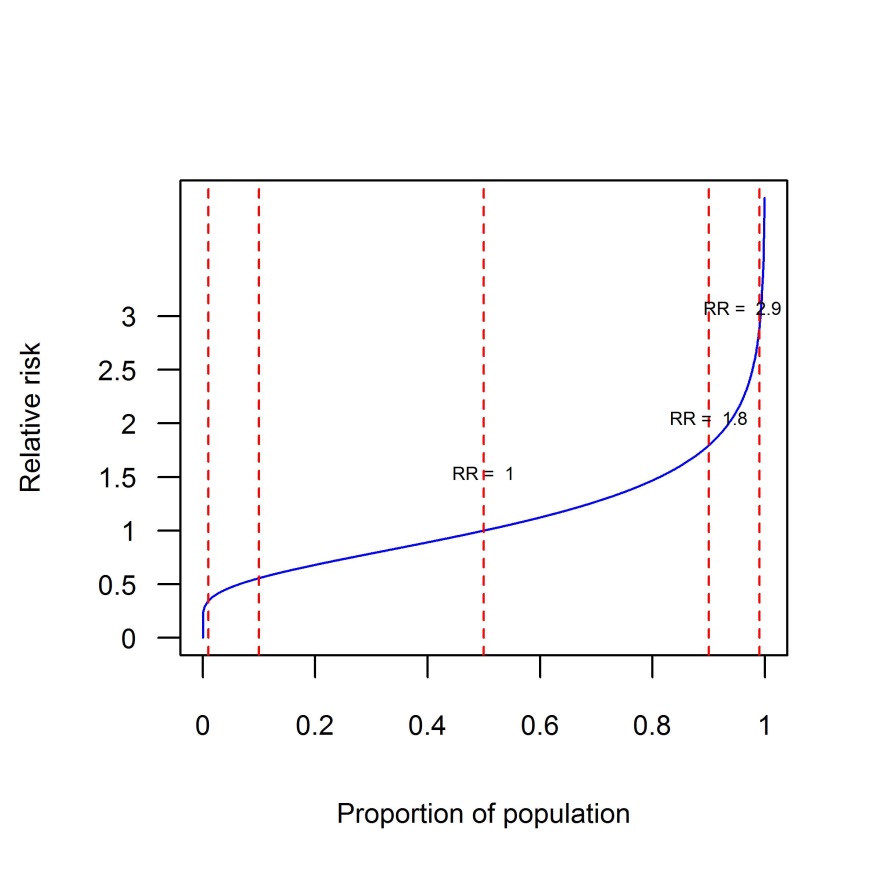
**

**Supplementary Figure 2: Population distribution of polygenic risk score (PRS) ordered by relative risk (RR) (compared with population median risk).** PRS is based on the 23 risk SNPs. Vertical red lines (left to right) correspond to 1%, 10%, 50%, 90%, and 99% centile, respectively.

**
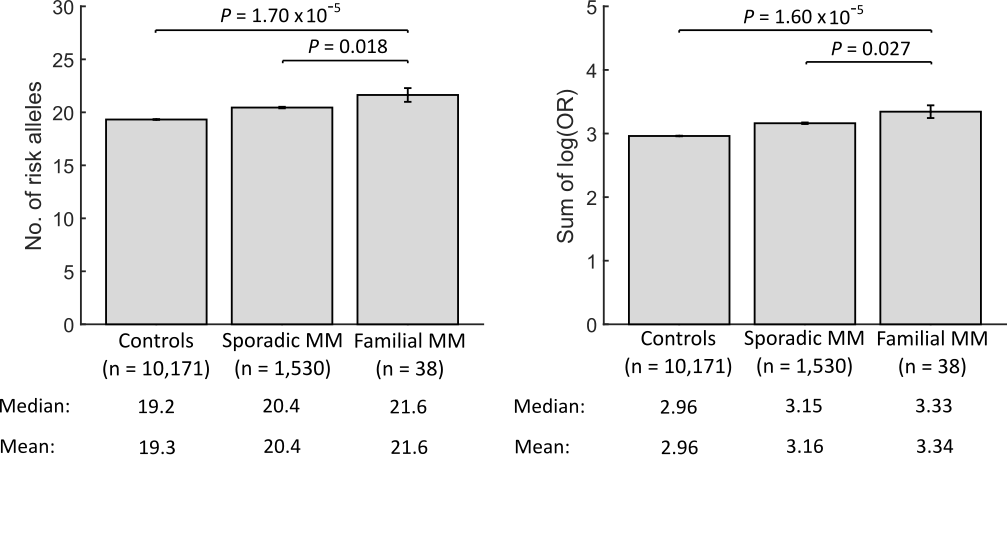
**

**Supplementary Figure 3: Polygenic risk scores (PRS) for familial MM, sporadic MM and population-controls.** A higher risk allele burden is seen in the familial MM compared with both sporadic MM and controls (difference in PRS score tested by one-sided Student’s t-test). (a) Based on number of risk alleles carried; (b) Calculated as the sum log-transformed odds ratios. The observed 1.08-fold enrichment of PRS in familial over sporadic cases is entirely compatible the expected familial risk attributable to the 23 risk SNPs of 1.10 given by:

$$\prod_{i}^{n=23} \frac{p_{i}r_{i}^{2}+q_{i}}{p_{i}r_{i}+q_{i}^{2}}$$

where *p_i_* is the frequency of the risk allele for locus *i*, *q_i_* = 1 − *p_i_*, and *r_i_* is the estimated per-allele OR.


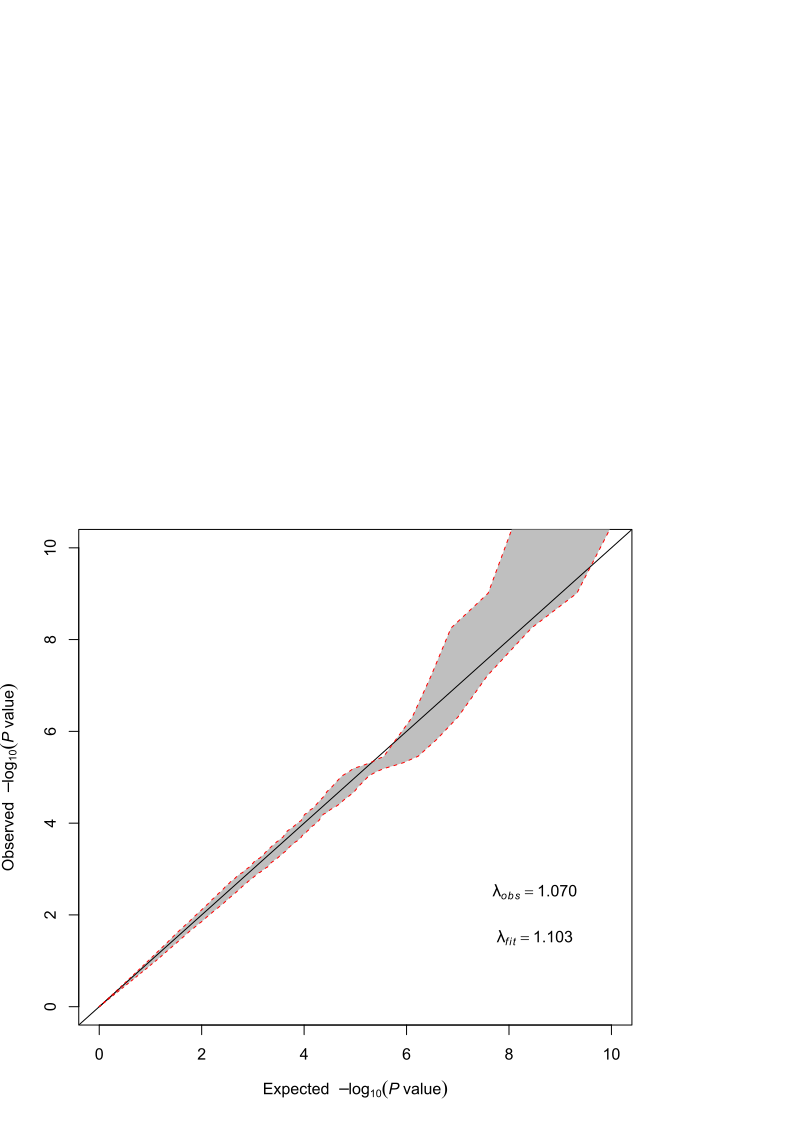


**Supplementary Figure 4: *Q-Q* plot comparing observed distributions of association statistics against those expected under a three-component model.** Grey shaded area represents the 80% confidence interval.


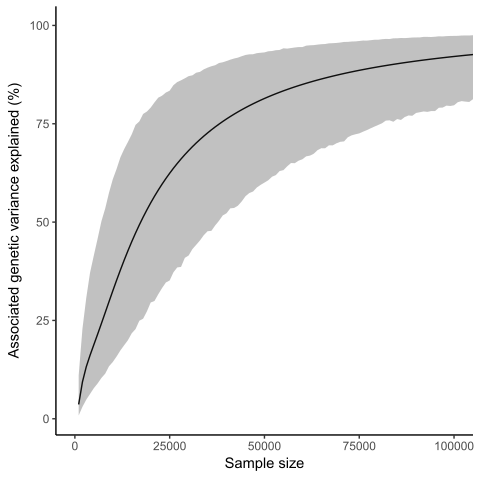


**Supplementary Figure 5: Projected percentage of GWAS heritability explained for a given sample size.** Results were obtained using a three-component model to estimate distribution of effect sizes. Grey shaded area represents the 95% confidence interval of the heritability estimate.


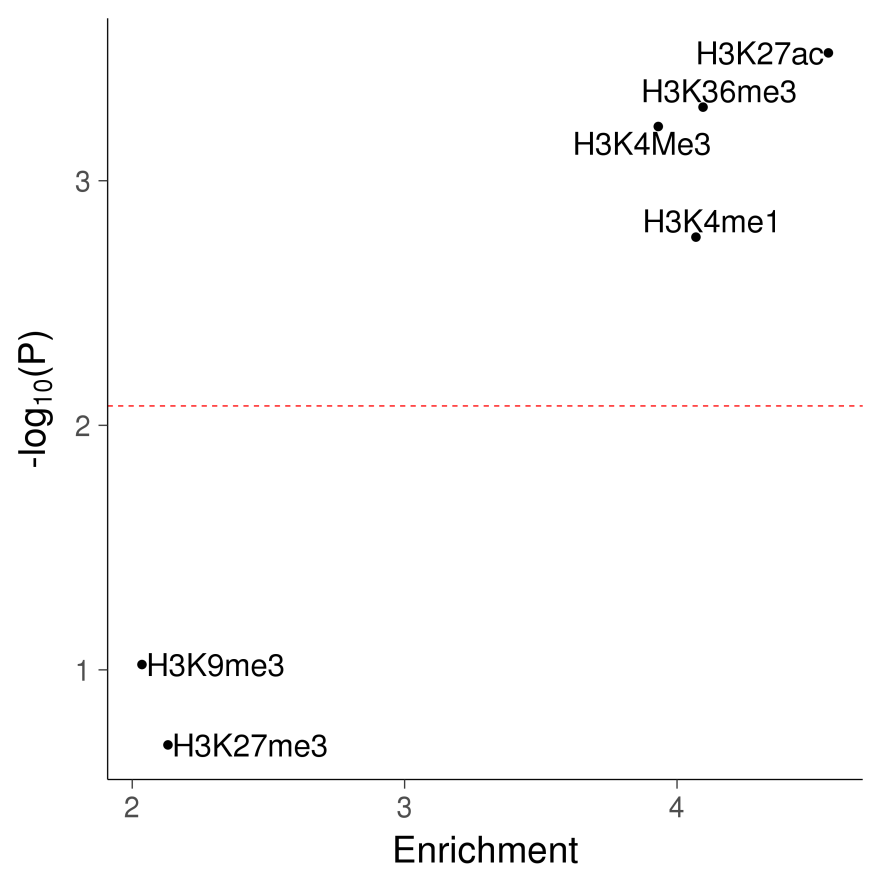

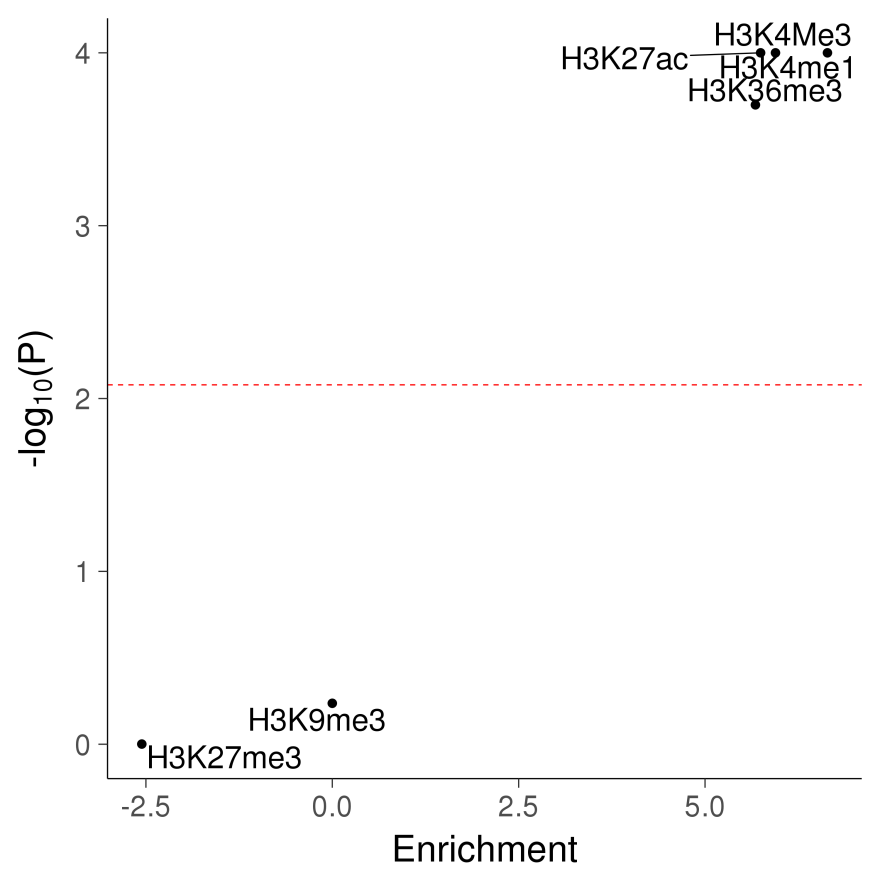


**a)**

**Supplementary Figure 6: The overrepresentation of histone marks in (a) naïve B and (b) KMS11 cells at the location of new and known MM risk SNPs demonstrates that risk SNPs are enriched in regions of open chromatin.** The red line denotes the Bonferroni corrected *P*-value threshold.

**b)**

**
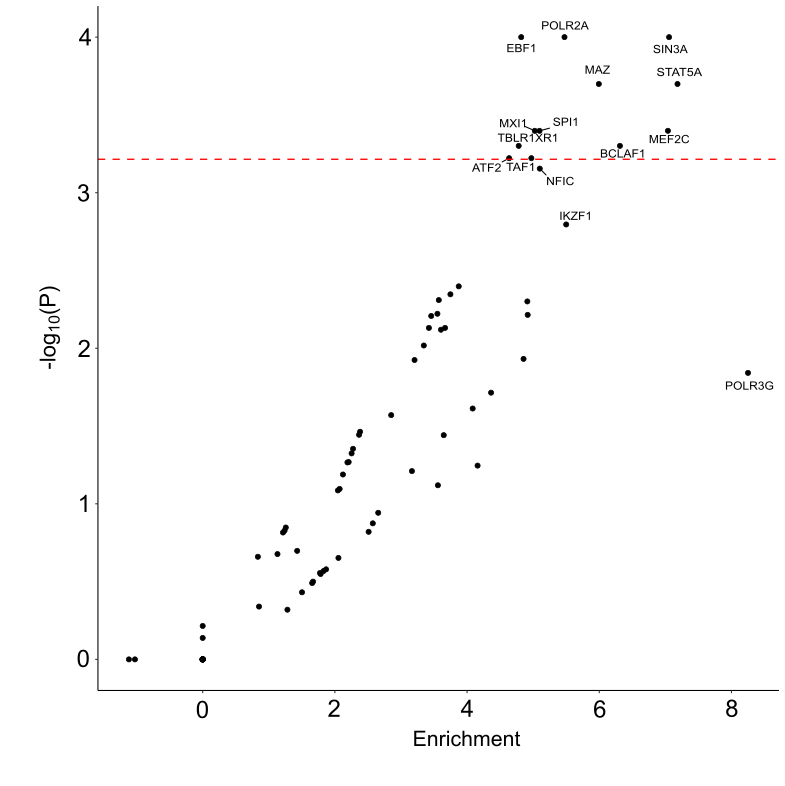
**

**Supplementary Figure 7: The overrepresentation of transcription factor (TF) binding sites in GM12878 cells at the location of new and known MM risk SNPs demonstrates that risk SNPs are enriched in regions of B-cell relevant TF binding.** The red line denotes the Bonferroni corrected *P*-value threshold.


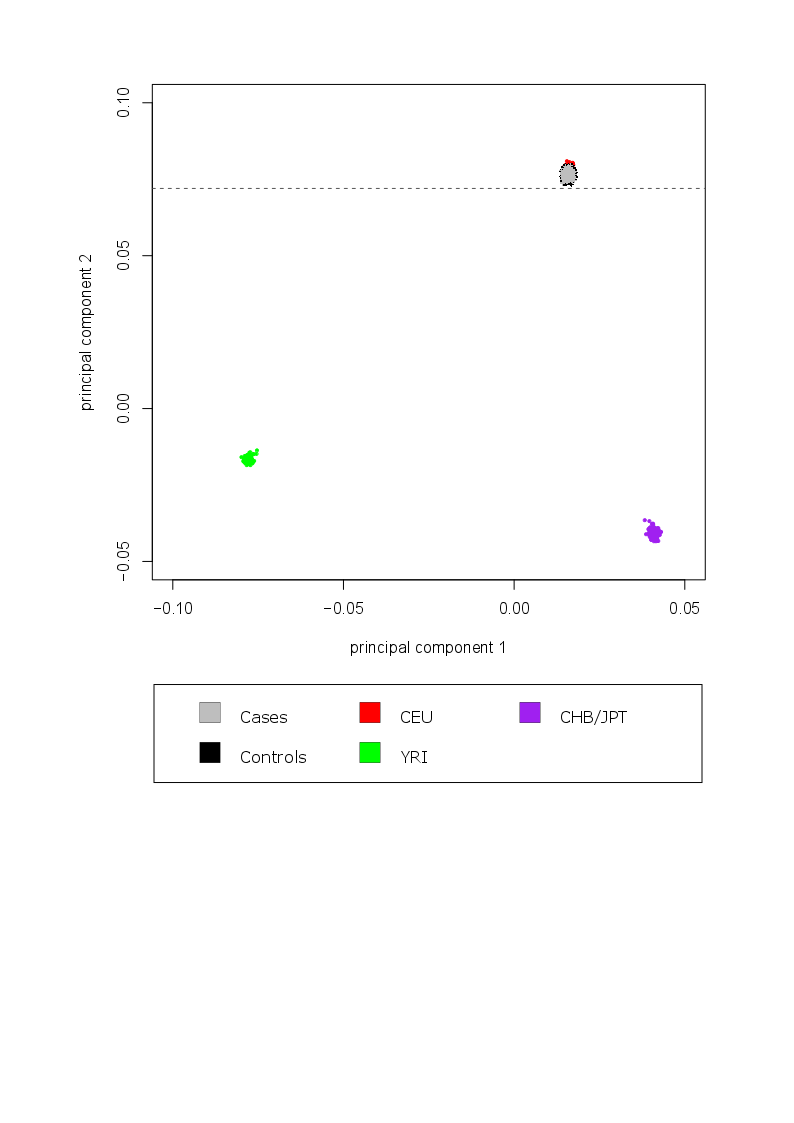


**Supplementary Figure 8: Principal components analysis plot for the OncoArray cohort after removal of non-European cases.** The first two principal components of the analysis are plotted. Cases and controls outside of the intervals 0.0155 ≤ *x* ≤0.019, and 0.0735 ≤ *y* ≤0.079 were excluded in order to remove individuals of non-European ancestry (grey dotted line shows the lower threshold of the second principal component). HapMap CEU individuals are plotted in red; CHB/JPT individuals are plotted in purple; YRI individuals are plotted in green. Cases are plotted in grey, controls plotted in black.


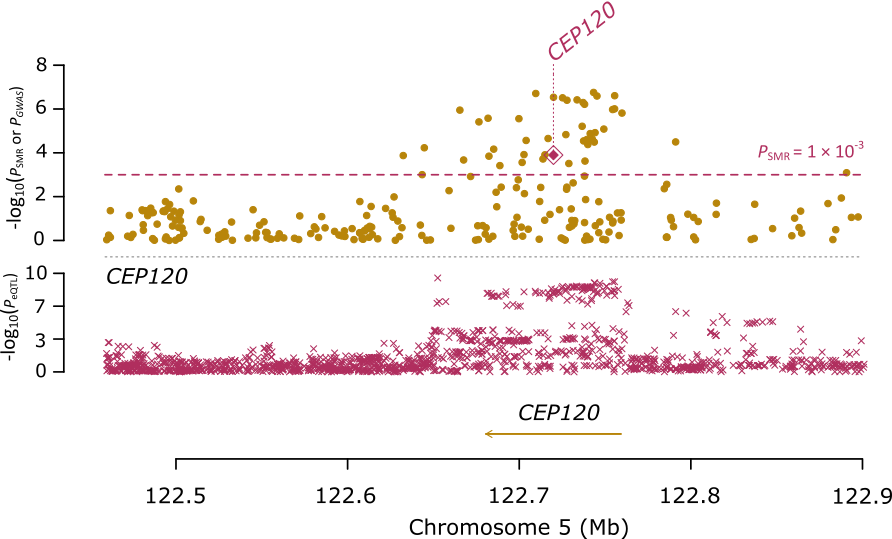


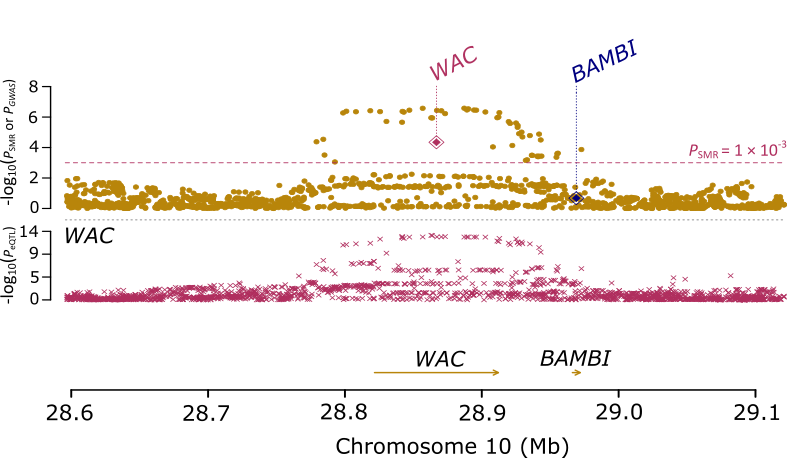


**a)**

**b)**

**Supplementary Figure 9: Summary data-based Mendelian Randomization (SMR) analysis locus plot at a) 5q23.2 and b) 10p12.1.** Upper panel - brown dots represent *P*-values for SNPs from the GWAS meta-analysis, diamonds represent *P-*values for probes from the SMR test; lower panel – crosses represent eQTL *P*-values of SNPs from MM plasma cells from 183 MRC MyIX trial patients (GEO: GSE21349) and 658 Heidelberg GMMG patients (EMBL-EBI: E-MTAB-2299), with genes passing the SMR (i.e. *P_SMR_* < 0.001) and HEIDI (*i.e*. *P_HEIDI_* > 0.05) tests highlighted in red. Probeset ID refers to Affymetrix U133 2.0 Plus Array custom chip definition file (CDF v.17) mapping to Entrez genes.


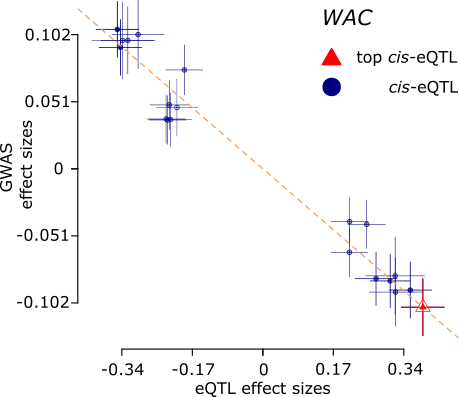

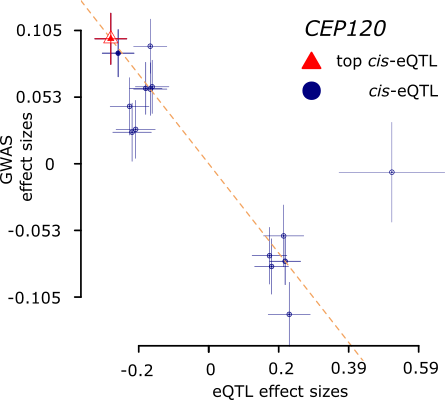


**b)**

**a)**

**Supplementary Figure 10: Summary data-based Mendelian Randomization analysis effect plot at (a) 5q23.2 and (b) 10p12.1** Blue dots represent effect sizes of SNPs from the GWAS meta-analysis against those from the eQTL study of MM plasma cells from 183 MRC MyIX trial patients (GEO: GSE21349) and 658 Heidelberg GMMG patients (EMBL-EBI: E-MTAB-2299). The top *cis*-eQTL is highlighted by a red diamond. Error bars are the standard errors of the SNP effects. An estimate of *b_xy_* at the top *cis*-eQTL is represented by the orange dotted line.


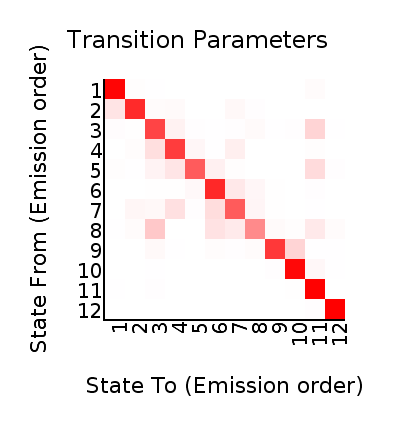

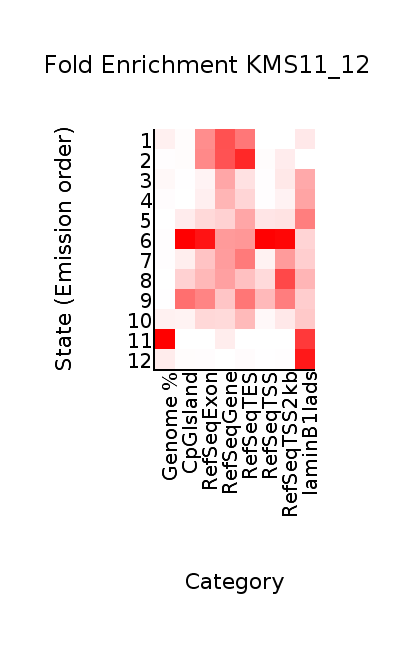

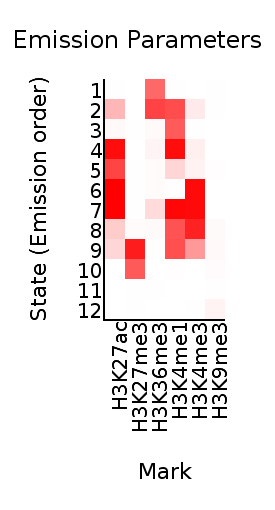


**Supplementary Figure 11: Heat maps outputted by ChromHMM pipeline show a) emission parameters, b) transition parameters and c) state functional enrichments for the KMS11 MM cell line.** Columns in **(c)** are labelled as follows: Genome % indicates the relative percentage of the genome represented by each state and relative fold enrichment for RefSeq transcription start sites (TSS); CpG Islands; 2000 base pair intervals around the TSS; exons; genes; transcript end sites (TES); evolutionary conservation; and nuclear lamina associated regions, respectively. Heat maps shown were used to assign states based on previously described rules^9-11^. The ChromHMM model was learned across 3 MM cell lines; JJN3, KMS11 and MM1S.

**REFERENCES**

1. Broderick, P. *et al.* Common variation at 3p22.1 and 7p15.3 influences multiple myeloma risk. *Nat Genet* **44**, 58-61 (2011).

2. Chubb, D. *et al.* Common variation at 3q26.2, 6p21.33, 17p11.2 and 22q13.1 influences multiple myeloma risk. *Nat Genet* **45**, 1221-1225 (2013).

3. Swaminathan, B. *et al.* Variants in ELL2 influencing immunoglobulin levels associate with multiple myeloma. *Nat Commun* **6**, 7213 (2015).

4. Mitchell, J.S. *et al.* Genome-wide association study identifies multiple susceptibility loci for multiple myeloma. *Nat Commun* **7**, 12050 (2016).

5. Chiecchio, L. et al. Deletion of chromosome 13 detected by conventional cytogenetics is a critical prognostic factor in myeloma. *Leukemia* **20**, 1610-7 (2006).

6. Neben, K. et al. Combining information regarding chromosomal aberrations t(4;14) and del(17p13) with the International Staging System classification allows stratification of myeloma patients undergoing autologous stem cell transplantation. *Haematologica* **95**, 1150-7 (2010).

7. Johnson, D.C. et al. Genome-wide association study identifies variation at 6q25.1 associated with survival in multiple myeloma. *Nat Commun* **7**, 10290 (2016).

8. de Souza, N. The ENCODE project. *Nat Methods* **9**, 1046 (2012).

9. Hoffman, M.M. *et al.* Integrative annotation of chromatin elements from ENCODE data. *Nucleic Acids Res* **41**, 827-41 (2013).

10. Fiziev, P. *et al.* Systematic Epigenomic Analysis Reveals Chromatin States Associated with Melanoma Progression. *Cell Rep* **19**, 875-889 (2017).

11. Schoenfelder, S. *et al.* Polycomb repressive complex PRC1 spatially constrains the mouse embryonic stem cell genome. *Nat Genet* **47**, 1179-1186 (2015).
