# Supplementary material for: Identification of multiple risk loci and regulatory mechanisms influencing susceptibility to multiple myeloma
Source: Nat Commun. 2018 Sep 13;9:3707. doi: 10.1038/s41467-018-04989-w (PMC6137048; doi:10.1038/s41467-018-04989-w)
Supplement: Supplementary file 4 — Description of Additional Supplementary Files [file 41467_2018_4989_MOESM4_ESM.pdf]

Description of Additional Supplementary Files

File Name: Supplementary Data 1
Description: Summary of functional annotation of the 23 risk loci. Newly identified risk loci are emboldened. 1 Where > 10 TF were implicated at a locus, only those that overlap with TF which demonstrated enrichment in GM12878 are shown here. A full list of TFs localising to loci are detailed in Supplementary Table 17.
